# A Descriptive Study of Psychiatric admissions of patients with Gambling Disorder at a tertiary hospital in the Philippines from the years 2016–2025

**DOI:** 10.1192/j.eurpsy.2026.10887

**Published:** 2026-07-31

**Authors:** C. L. Z. Chua, A. K. L. Enriquez

**Affiliations:** Department of Psychiatry, The Medical City, Pasig City, Philippines

## Abstract

**Introduction:**

Gambling is a deeply rooted cultural activity in the Philippines as a form of recreation, and a a significant portion of the population engages in gambling activities, however, specific data on the prevalence of Gambling Disorder is are limited due to the lack of comprehensive studies. Psychiatric admissions specifically with Gambling Disorder remain relatively less studied and under-investigated.

**Objectives:**

This study aims to determine the profile of patients admitted for Gambling Disorder admitted to a tertiary psychiatric facility in the Philippines from 2016-2025, to describe the demographic characteristics, types of gambling, and the associated factors with the addiction.

**Methods:**

The study involved a retrospective review of medical charts, focused on sociodemographic factors, prior psychiatric admissions, and family history of substance use and related disorders, as well as clinical profiles including co-morbid psychiatric disorders. Descriptive statistical methods were used to analyze the findings.

**Results:**

48 patients were diagnosed with Gambling Disorder were admitted to the psychiatric facility between 2016 and 2025. The number of admissions increased in recent years from 2023-2025. Most patients were male (89.6%), mainly between 21 and 50 years old. A majority were married (52.1%) or single (43.8%), and more than half had completed college education (56.3%). Casino gambling was the most common form of gambling 64.6%, followed by online gaming at 33.3%. Many patients had co-morbid psychiatric conditions, with Mood disorders affecting 79.2% and Substance-related disorders present in 62.5%. Substance use involved methamphetamine, alcohol, cannabis, benzodiazepines, and opioids, while 35.4% reported use of multiple substances. Additional co-morbidities included Psychotic disorders (12.5%), Personality disorders (12.5%), and Neurodevelopmental disorders (14.6%). Almost half of the patients (45.8%) had previous psychiatric admissions, and 27.1% had a family history of substance-related disorders.

**Conclusions:**

A significant proportion of patients with Gambling Disorder had co-morbid psychiatric conditions highlighting the complex clinical profile of these patients, which emphasizes the need for comprehensive and integrated treatment approaches. These findings suggest that Gambling Disorder is an emerging public health issue in the Philippines that requires targeted intervention, increased awareness, and specialized services within psychiatric care settings. Further studies could investigate long-term outcomes and treatment approaches for this population.

**Disclosure of Interest:**

None Declared

